# Depletion of ID3 enhances mesenchymal stem cells therapy by targeting BMP4 in Sjögren’s syndrome

**DOI:** 10.1038/s41419-020-2359-6

**Published:** 2020-03-05

**Authors:** Lei Hu, Junji Xu, Tingting Wu, Zhipeng Fan, Lingyun Sun, Yi Liu, Yan Li, Chunmei Zhang, Jingsong Wang, Yaozhong Ding, Songlin Wang

**Affiliations:** 1 Salivary Gland Disease Center and Beijing Key Laboratory of Tooth Regeneration and Function Reconstruction, Capital Medical University School of Stomatology, Beijing, 100050 China; 2 National Institute of Dental and Craniofacial Research, National Institutes of Health, Bethesda, MD 20892 USA; 3 Department of Rheumatology and Immunology, Affiliated Drum Tower Hospital of Nanjing University Medical School, Nanjing, China; 4 Health Management Center, The Third Xiangya Hospital of Central South University, Changsha, China; 5 Fortune Link Triones (Beijing) Scitech Co., Ltd., Beijing, China; 6 Department of Biochemistry and Molecular Biology, Capital Medical University School of Basic Medical Sciences, Beijing, 100069 China; 7 Department of Immunology, Capital Medical University, Beijing, 100069 China

**Keywords:** Immunological disorders, Mesenchymal stem cells

## Abstract

Mesenchymal stem cell (MSCs) transplantation has been used to treat Sjögren’s syndrome (SS) based on the immunoregulatory properties of MSCs. However, the effectiveness need improving and its underlying intrinsic mechanisms remain largely unknown. Here, we show that *Id3* is upregulated in bone marrow-derived MSCs (BMMSCs) isolated from NOD/ShiLtJ mice, a widely used SS model, compared with ICR mice as control, suggesting that it functions in SS development and therapy. Transplantation of *Id3*-deficient BMMSCs rescues salivary gland function more effective than wild-type BMMSCs in NOD/ShiLtJ mice. Mechanistically, we show that ID3 negatively regulated BMP4 expression by preventing binding of basic helix–loop–helix protein E2A to the promoter of the *Bmp4* gene. BMP4 in turn promoted PGE2 production in MSCs, and exhibited enhanced suppressive activities of T-cell proliferation and Th1 differentiation. Importantly, BMMSCs from SS patients showed significantly lower BMP4 and PGE2 expression than those from healthy individuals. Taken together, our findings revealed the targeting Id3 may be therapeutically useful for improving MSC immunoregulation and effectiveness of MSCs therapy for SS.

## Introduction

Sjögren’s syndrome (SS) is a chronic autoimmune disorder that typically impairs the function of exocrine glands, mainly the salivary and lacrimal glands, resulting in symptoms of dry mouth and eyes^1^. Dysfunction of immune systems have been shown to play a significant role in SS pathogenesis, including innate immune system, B­ cell activation, and resultant activation of T cells—mainly type 1 T­helper (Th1) cells. Current treatments for SS patients include steroids and nonsteroidal anti-inflammatory drugs, which relive symptoms, but do not cure the disease and have serious side effects with long-term use. Mesenchymal stem cells (MSCs) have immunoregulatory properties and show therapeutic effects on experimental and clinical autoimmune diseases and inflammation, including SS, Crohn’s disease, systemic lupus erythematosus, and rheumatoid arthritis^2–5^. However, the clinical trial showed the efficacy MSC-based therapies are not consistent, partial patients present useless^6–8^. Thus, uncovering the mechanisms that maintain MSCs immunoregulatory properties or MSC-based therapeutic effects is important for improving MSC-based therapies.

Inhibitor of DNA binding proteins (IDs), including ID1-4, are helix–loop–helix (HLH) transcription factors that lack the basic region required for DNA binding found in other HLH proteins^9^. IDs bind to basic (b) HLH E proteins and disrupt the formation of dimers, thus inhibiting DNA binding and transcriptional activity. E proteins have been found to play key roles in the differentiation and biological properties of various cells. Subsequent studies have uncovered E proteins inhibit cell cycle progression and implicated in cellular senescence activate through activate transcription of the CDK inhibitors^10^. As key regulators of E proteins, ID proteins have been implicated in the proliferation and self-renewal of embryonic stem cells, somatic stem cells, progenitors and hematopoietic stem cells^11–14^.

ID3 contributes to cellular differentiation and proliferation. It is involved in T-cell receptor (TCR)-mediated T-cell development^15,16^ and in the differentiation of regulatory T cells (Tregs), Th17 cells, Th9 cells, and B cells^17–19^. *Id3*^*−/−*^ mice develop many symptoms similar to those found in Sjögren’s syndrome, like immune cells chronically attack the lachrymal and salivary glands, resulting in impaired tear and saliva secretion. T-cell dysfunction has a prominent role in the development of SS in mice that lack *Id3* (*Id3*^*−/−*^)^20,21^. However, the roles of ID3 in mediating the immunoregulatory function of MSCs in the development of SS is yet unknown.

We found that Id3 was upregulated in Bone marrow-derived MSCs (BMMSCs) derived from NOD/ShiLtJ mice, which is SS model, when compared with ICR mice. Depletion of Id3 (*Id3*^*−*/*−*^) enhanced the inhibiting function of BMMSCs on T-cell proliferation and IFN-gamma production in vitro and in vivo. In this study, we showed that ID3 controls the immunosuppressive functions bone marrow MSCs by inhibiting BMP4 expression, which decreasing PGE2 production. Our findings provide novel insights into the immunoregulatory functions of MSCs in chronic inflammatory or autoimmune diseases.

## Results

### Deletion of *Id3* enhanced immunosuppression of BMMSCs to T-cell proliferation and IFN-γ production in vitro and in vivo

**Fig. 1:**
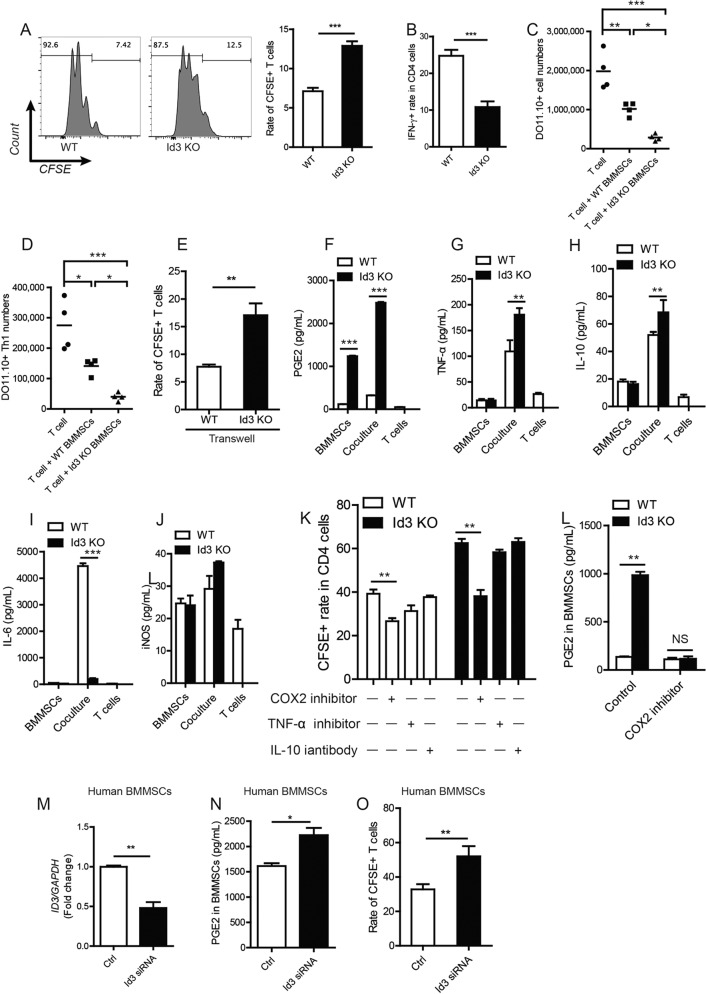
*Id3*^*−*/*−*^ BMMSCs enhanced immunosuppression of *Id3*-deficient BMMSCs to T-cell proliferation and IFN-gamma production in vitro and in vivo. **a** CFSE analysis of cell proliferation for T cells cultured with bone marrow-derived mesenchymal stem cells (BMMSCs) form WT mice and *Id3*^−*/*−^ mice (WT and Id3 KO). **b** Frequencies of IFN-γ producing Th1 cell subsets in the presence of *Id3*^−*/*−^ BMMSCs or WT BMMSCs; WT or *Id3*^−*/*−^ BMMSCs and CD4^+^ T cells derived from DO11.10 TCR-transgenic mice were mixed at a ratio of 1:2 and injected into BALB/cJ mice. **c**, **d** Flow cytometric analyses showing changes in CD4^+^ T and Th1 cell number in BALB/cJ mice injected with CD4^+^ T cells from DO11.10 TCR-transgenic mice combined with *Id3*^−*/*−^ or WT BMMSCs and a subsequent ovalbumin challenge, compared with T cells only. **e** T-cell proliferation when cocultured with WT or *Id3*^−*/*−^ BMMSCs in Transwell plates. **f–j** Changes in PGE_2_, TNF-α, IL-10, IL-6 and inducible nitric oxide synthase (iNOS) expression in supernatants of WT or *Id3*^−*/*−^ BMMSCs, T cells and cocultures. **k** Effect of COX2 IL-10, TNF-α or IL-10 inhibition on immunosuppressive effects of WT and *Id3*^−*/*−^ BMMSCs. **l** PGE_2_ secretion in response to COX2 inhibition. **m** ID3 expression, as assessed by Western blot, in human BMMSCs transfected with *ID3*-specific siRNA or siRNA only (Ctrl). **n** PGE_2_ secretion, as assessed by ELISA, in control and *ID3*-knockdown human BMMSCs. **o** CFSE analyses of cell proliferation in T cells cultured with *ID3*-knockdown human BMMSCs versus control BMMSCs. Values are means ± SD (*n* = 5, three independent experiments). Student’s t tests and one-way ANOVA. *P ≤ 0.05; **P ≤ 0.01; ***P ≤ 0.001.

Firstly, we compared gene expression in BMMSCs between NOD/ShiLtJ mice, which is widely used SS model, and ICR mice as control. Among the reported SS related genes^22^, *Id3* is the highest expressed gene in NOD/ShiLtJ mice (Supplementary Fig. 1). We generate *Id3* knockout (*Id3*^*−/−*^) mice and found BMMSCs from wild-type (WT) and *Id3*^−*/−*^ mice exhibited no significant differences in proliferation and apoptosis (Supplementary Fig. 2B, C). To identify the role of *Id3* on immunoregulation function of MSCs, normal splenic CD4^+^CD25^*−*^ naive T cells were cocultured with WT or *Id3*^*−/*−^ BMMSCs in the presence of anti-CD3 and anti-CD28 antibodies. T cells cultured with *Id3*^*−/−*^ BMMSCs showed much lower proliferative responses, as determined by carboxyfluorescein succinimidyl ester-dilution assays, than T cells cultured with WT BMMSCs (Fig. 1a, and Supplementary Fig. 2D, E). No significant differences were found in T-cell apoptosis between WT and *Id3*^*−*/*−*^ BMMSCs cultures (Supplementary Fig. 3A, B). Moreover, *Id3*^*−/−*^ BMMSCs treatment decreased the frequency of interferon (IFN)-γ producing Th1 cells compared with WT BMMSCs treatment (Fig. 1b), but did not change the frequency of interleukin (IL)-4 producing Th2 cells and Foxp3^+^ Tregs (Supplementary Fig. 3C–F). These results indicated that the immunosuppressive function of BMMSCs was enhanced in the absence of *Id3* in vitro.

Next, WT or *Id3*^*−/−*^ BMMSCs and CD4^+^ T cells (KJ-126^+^) derived from DO11.10 TCR-transgenic mice were mixed and injected into syngeneic BALB/cJ mice, who were then challenged by ovalbumin protein (peptide) administered by intravenous injection. Seven days later, flow cytometric analysis showed that there were fewer CD4^+^KJ-126^+^ T cells in *Id3*^*−/−*^ BMMSC-injected BALB/cJ mice than in WT BMMSC-injected mice (Fig. 1c), and fewer Th1 cells within the injected CD4^+^ T-cell population (Fig. 1d). However, there were no changes in the number of Th2 cells or Tregs between these two groups (Supplementary Fig. 3G–K). Thus, *Id3*^*−/−*^ BMMSCs also inhibited T-cell growth and Th1 cell differentiation to specific antigen stimulation in vivo. These results indicated that the immunosuppressive function of *Id3*^*−/−*^ BMMSCs was enhanced in vitro and in vivo.

To elucidate whether *Id3*^*−/−*^ BMMSC-mediated immunosuppressive effects on T cells requires direct cell contact, we cultured normal CD4^+^ T cells with BMMSCs in a Transwell plate. T cells cultured with *Id3*^*−/−*^ BMMSCs showed much lower proliferative responses than T cells cultured with WT BMMSCs (Fig. 1e), suggesting that the inhibitory function of *Id3*^*−/−*^ BMMSCs occurs through secretion of cytokines or other soluble factors. Accordingly, we examined the expression of a panel of MSC-related immunoregulatory mediator and found that prostaglandin E_2_ (PGE_2_), IL-10, and tumor necrosis factor (TNF)-α were upregulated in the supernatants of *Id3*^*−/−*^ BMMSCs and T-cell cocultures, whereas IL-6 was downregulated (Fig. 1f–j). Among the upregulated immunoregulatory mediator, PGE_2_ showed the most significant increase (10-fold) in *Id3*^*−/−*^ BMMSC and T-cell cocultures. We attributed this increased PGE_2_ expression to the *Id3*^*−/−*^ BMMSCs, because *Id3*^*−/−*^ BMMSCs monoculture (without T cells) also produced significantly higher levels of PGE_2_ than WT BMMSCs (Fig. 1f). In contrast, TNF-α and IL-10 levels did not differ between *Id3*^*−/−*^ and WT BMMSCs monocultures (Fig. 1g, h). Importantly, inhibition of the activity of PGE_2_ with celecoxib, a cyclooxygenase 2 (COX2) inhibitor, markedly decreased the inhibitory effect of *Id3*^*−/−*^ BMMSCs on T-cell proliferation; but that was not the case when TNF-α inhibitor pomalidomide^22,23^ or an anti-IL-10 antibody was used (Fig. 1k, l, and Supplementary Fig. 3L). These results indicated that the enhanced immunosuppressive functions of *Id3*^*−/−*^ BMMSCs can be mainly attributed to PGE_2_. The effects of ID3 on immunosuppression in MSCs were also verified in human BMMSCs from healthy volunteers. Knockdown of *ID3* in human BMMSCs by *ID3*-specific small interfering RNA (siRNA; Fig. 1m) resulted in higher levels of PGE_2_ compared with control BMMSCs (Fig. 1n), and suppression of T-cell proliferation (Fig. 1o).

### Deletion of *Id3* increases prostaglandin E2 (PGE2) secretion in BMMSCs through BMP4

**Fig. 2:**
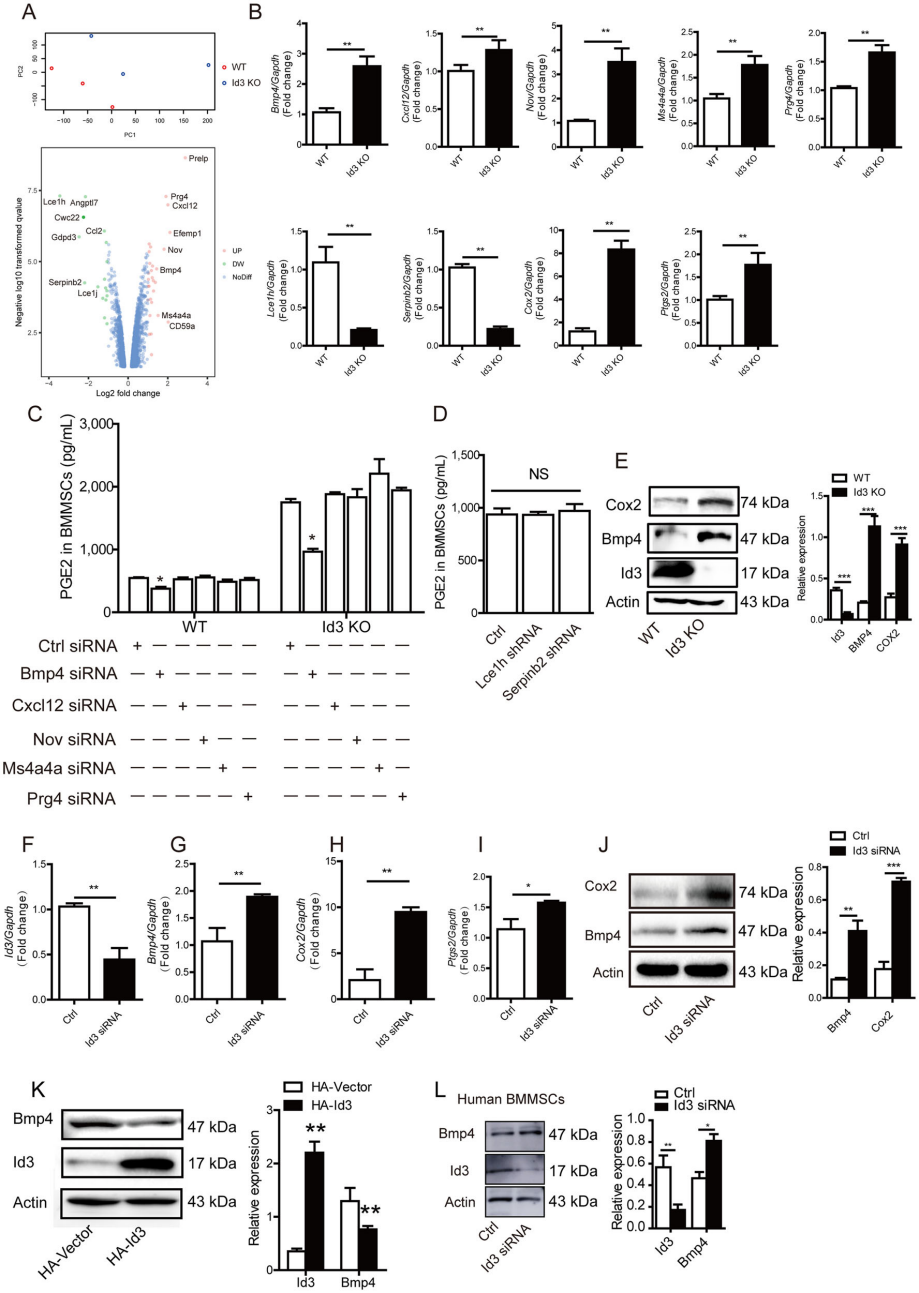
Deletion of *Id3* increases prostaglandin E2 (PGE2) secretion in BMMSCs through BMP4. **a** Principal components analysis (PCA) and volcano graphic showing differences in gene expression between bone marrow-derived mesenchymal stem cells (BMMSCs) form WT mice and *Id3*^−*/*−^ mice (WT and Id3 KO), analyzed by Affymetrix gene chip arrays (*n* = 3 per group). **b** Differentially expressed genes were analyzed by qPCR. **c** Effects of *Bmp4, Cxcl12, Nov, Ms4a4a, Prg4* knockdown by siRNA on prostaglandin E_2_ (PGE_2_) secretion levels in in supernatant from WT BMMSCs and *Id3*^−/−^ BMMSCs; negative siRNA was transfected as control group (Ctrl). **d** Effects of *Lce1h* and *Serpinb2* knockdown by shRNA on PGE_2_ levels in WT BMMSCs. **e** Western blot of ID3, BMP4, and COX2 expression in WT and *Id3*^−/−^ BMMSCs. **f**–**i** Effect of *Id3* siRNA transfection of WT BMMSCs on *Id3*, *Bmp4*, *Cox2*, and *Ptgs2* mRNA levels, analyzed by qPCR. **j** Western blot of BMP4 and COX2 levels in *Id3* siRNA and control group. **k** Western blot of BMP4 level after transfection of WT BMMSCs with *Id3* overexpressing viral vector; blank vector was transfected as control group. **m** Western blot of BMP4 expression in human BMMSCs transfected with *ID3* siRNA. Values are means ± SD (*n* = 5, three independent experiments). Student’s *t* tests and one-way ANOVA. *P ≤ 0.05; **P ≤ 0.01; ***P ≤ 0.001.

To investigate the molecular mechanisms through which ID3 regulates PGE_2_-mediated immunosuppression of BMMSCs, we compared functional gene expression changes between WT and *Id3*^*−/−*^ mouse BMMSCs using the Affymetrix gene chip array analysis and identified several differentially expressed genes (Fig. 2a), among the 1320 upregulated genes and 1001 downregulated genes in *Id3*^*−/−*^ MSCs, respectively (Supplementary Table). Quantitative real-time (qPCR) showed that *Bmp4*, *Cxcl12*, *Nov*, *Ms4a4a*, *Prg4*, *Cox2*, and *Ptgs2* were upregulated, whereas *Lce1h* and *Serpinb2* were downregulated in *Id3*^*−/−*^ BMMSCs compared with WT BMMSCs (Fig. 2b). We knocked down *Bmp4*, *Cxcl12*, *Nov*, *Ms4a4a*, and *Prg4* using siRNA (Supplementary Fig. 4A) in *Id3*^*−/−*^ and WT BMMSCs, and *Lce1h* and *Serpinb2* using short hairpin RNA (Supplementary Fig. 4B) in WT BMMSCs. Only knockdown of *Bmp4* significantly suppressed the upregulation of PGE_2_ in *Id3*^*−/−*^ BMMSCs, although PGE_2_ expression was still higher than in WT BMMSCs (Fig. 2c, d). Reduction of *Bmp4* also reduced PGE_2_ levels in WT BMMSCs (Fig. 2c). Consistent with these findings, western blot analyses confirmed that BMP4 and COX2 proteins were significantly upregulated in *Id3*^*−/−*^ BMMSCs compared with WT BMMSCs (Fig. 2e). ID3’s role in MSC immunoregulation by targeting BMP4 was validated by knocking down *Id3* in WT BMMSCs with siRNA in vitro (Fig. 2f–l, and Supplementary Fig. 4C–F). Thus, increased PGE_2_ production in *Id3*^*−/−*^ BMMSCs was driven by BMP4.

### ID3 regulates BMP4 by preventing E2A binding to *Bmp4* promoter

**Fig. 3:**
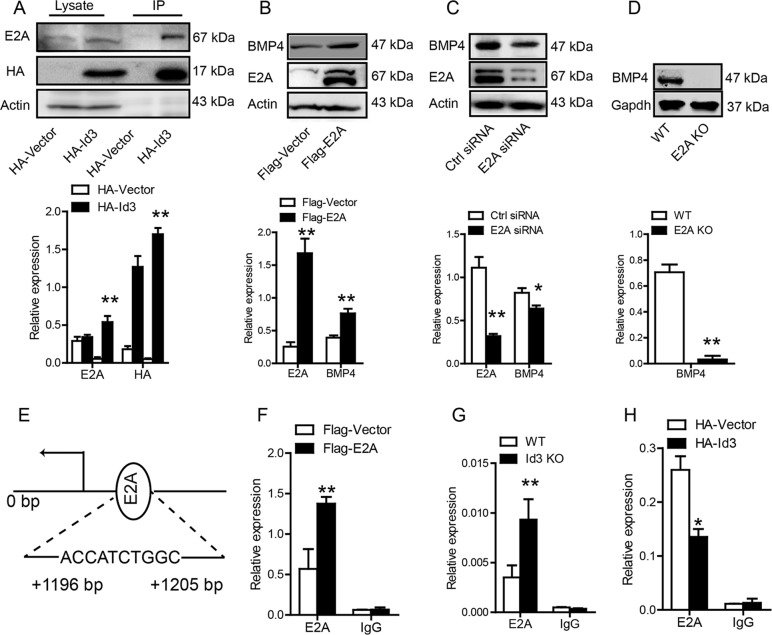
ID3 regulates BMP4 by preventing E2A binding to *Bmp4* promoter. **a** Co-immunoprecipitation analyses of binding of ID3 to E2A in bone marrow-derived mesenchymal stem cells (BMMSCs). **b** Western blot analyses the BMP4 expression after E2A overexpression in BMMSCs. **c** Western blot analyses the BMP4 expression after E2A knockdown by E2A siRNA in BMMSCs. **d** Western blot analyses the BMP4 expression in BMMSCs from E2A^−/−^ mice. **e** Prediction of E2A binding site in the *Bmp4* promoter. **f**–**h** Chromatin immunoprecipitation analyses of E2A binding after *Id3* knockdown (BMMSCs from WT mice and *Id3*^−*/*−^ mice), *Id3* overexpression (HA labeled vector and Id3 transfected WT BMMSCs), and E2A overexpression (Flag labeled vector and E2A transfected WT BMMSCs). Values are means ± SD (*n* = 3, three independent experiments). Student’s *t* tests and one-way ANOVA. *P ≤ 0.05; **P ≤ 0.01.

ID3 regulate target genes by inhibiting E2A formation of heterodimers^24^. Co-immunoprecipitation analyses showed that ID3 bound to E2A in WT BMMSCs (Fig. 3a). Overexpression of E2A upregulated BMP4 and COX2 levels in BMMSCs (Fig. 3b). Conversely, reduction of E2A expression using siRNA in BMMSCs blocked the expression of BMP4 (Fig. 3c). Furthermore, analysis of E2A-deficient BMMSCs from *E2a* knockout mice (E2a^f/f^Heb^f/f^ER-Cre^+^ mice)^25^ revealed a significant reduction in BMP4 protein expression (Fig. 3d). Chromatin immunoprecipitation assays found that E2A bound to the *Bmp4* promoter in BMMSCs (Fig. 3e–h). Overexpression of E2A enhanced the binding of E2A to the *Bmp4* promoter in WT BMMSCs (Fig. 3f). Deletion of *Id3* enhanced E2A binding to the *Bmp4* promoter in *Id3*^*−/−*^ BMMSCs (Fig. 3g), and overexpression of *Id3* reduced binding of E2A to the *Bmp4* promoter in WT BMMSCs (Fig. 3h).

### BMP4 mediate BMMSCs therapy for SS

**Fig. 4:**
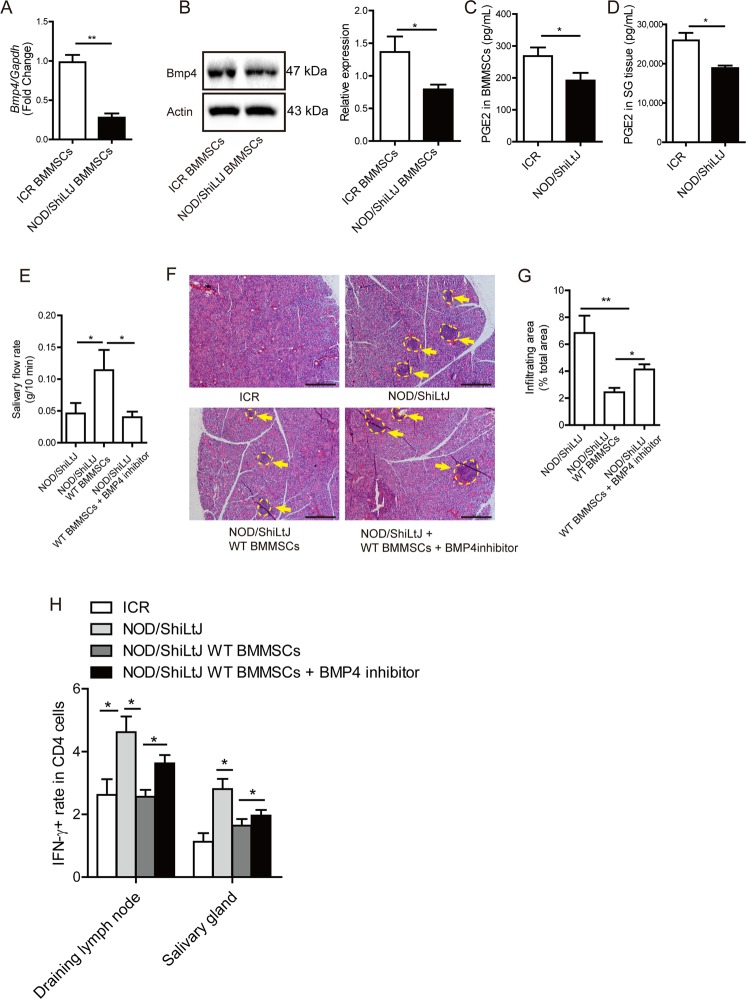
Effects of BMP4 on the therapeutic efficacy of BMMSCs in SS. **a**, **b** BMP4 mRNA and protein levels in bone marrow-derived mesenchymal stem cells (BMMSCs) derived from NOD/ShiLtJ and ICR mice. **c**, **d** Prostaglandin E_2_ (PGE_2_) levels in supernatant of BMMSCs and submandibular glands from NOD/ShiLtJ and ICR mice. **e** The saliva flow rate of WT mice BMMSCs-treated mice was significantly blocked with BMP4 inhibition. **f**, **g** H&E staining showed that the area of infiltrating in the submandibular glands of NOD/ShiLtJ mice following BMMSCs injection was significantly smaller than untreated NOD/Ltj mice, and this protective effect was significantly decreased with BMP4 inhibition. Yellow dotted and arrow showed area represent infiltrating area in the submandibular glands. **h** IFN-γ^+^ T cells in the submandibular glands from BMP4 BMMSCs injection inhibition were upregulated compared with BMMSCs injection group (*n* = 5, three independent experiments). Values are means ± SD. Student’s t tests and one-way ANOVA. *P ≤ 0.05; **P ≤ 0.01, scale bar: 200 μm.

In BMMSCs from NOD/ShiLtJ mice, BMP4 expression was significantly decreased compared with those from ICR mice (control mice) at both mRNA and protein levels (Fig. 4a, b). PGE_2_ levels were decreased in BMMSCs and submandibular glands from NOD/ShiLtJ mice compared with those from ICR mice (Fig. 4c, d). BMMSCs transplantation increases the salivary flow rate and reduced the area of inflammation in the submandibular glands, and decreased IFN-γ production T (Th1) cells in the submandibular glands. The therapeutic effects of allogeneic BMMSCs transplantation on NOD/ShiLtJ mice were abolished by inhibition of BMP4 with a specific inhibitor before the WT BMMSCs were transplanted (Fig. 4e–h).

### *Id3*^*−/−*^ BMMSCs suppress SS symptom more efficiently than WT BMMSCs in NOD/ShiLtJ mice

**Fig. 5:**
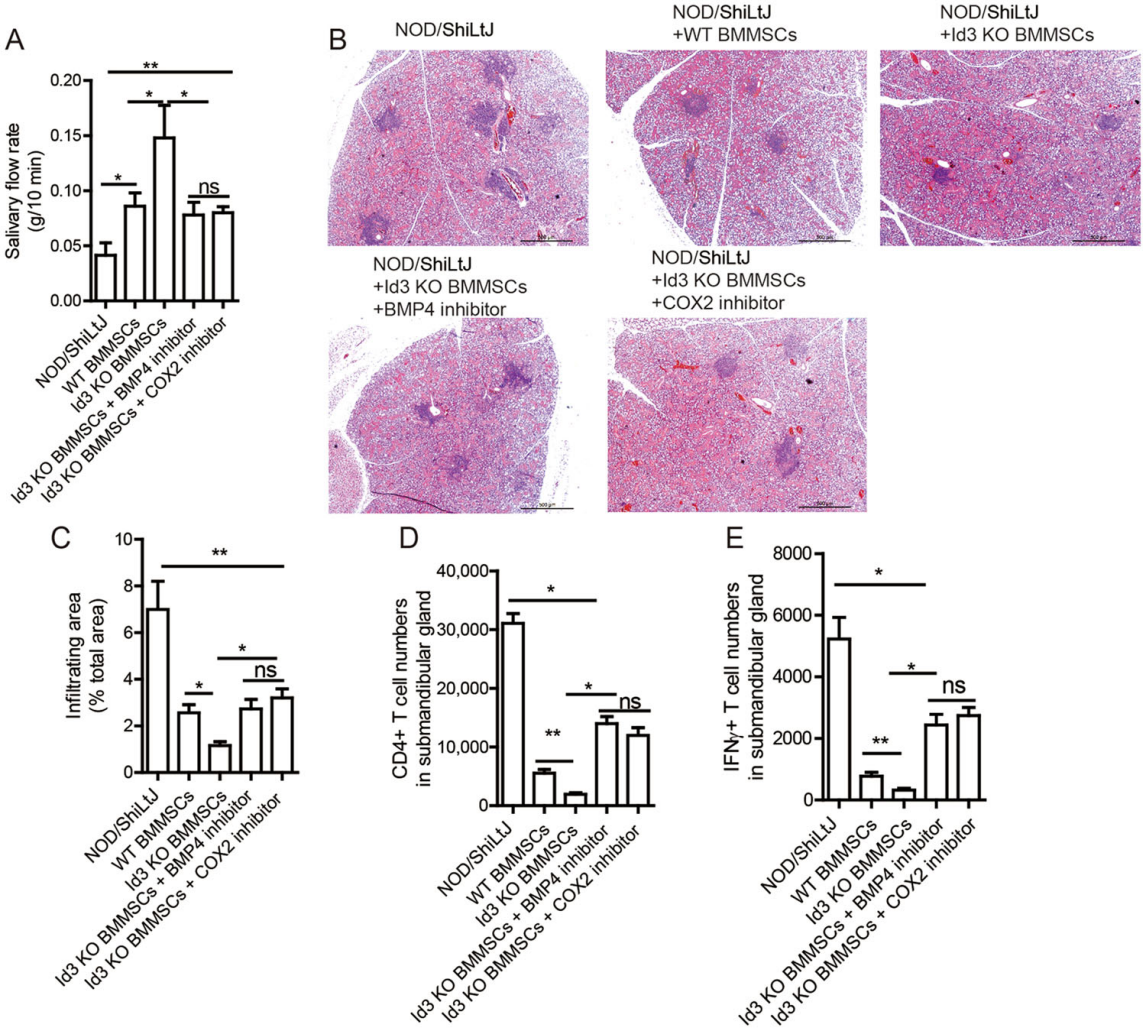
*Id3*^−*/*−^ BMMSCs suppress SS more efficiently than WT BMMSCs. **a** Salivary flow rates in *Id3*^−*/*−^ BMMSC- (with/without previous BMP4 or COX2 inhibition) and WT BMMSC-treated NOD/ShiLtJ mice. **b**, **c** H&E staining of inflammatory cell infiltration in submandibular glands and effects of BMP4 inhibition; scale bars: 200 μm. **d**, **e** Number of CD4^+^ T cells and Th1 cells in submandibular glands (*n* = 10, three independent experiments). Values are means ± SD. Student’s *t* tests and one-way ANOVA. *P ≤ 0.05; **P ≤ 0.01.

**Fig. 6:**
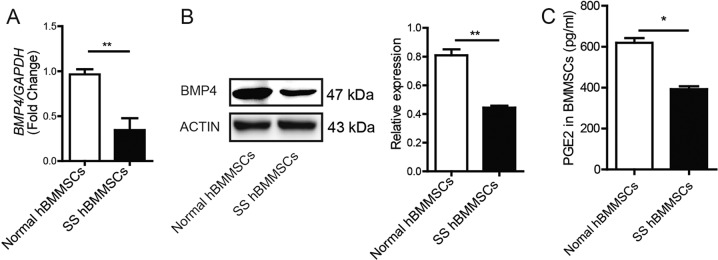
BMP4 and PGE2 decreased in SS patient’s BMMSCs. **a** BMP4 mRNA expression in BMMSCs from patients with primary Sjögren’s syndrome (pSS) and healthy volunteers. **b** Western blot of BMP4 expression in BMMSCs from pSS and healthy volunteers. **c** PGE_2_ levels in supernatant of BMMSCs from healthy volunteers and SS patients (*n* = 5, three independent experiments). Values are means ± SD. Student’s *t* tests and one-way ANOVA. *P ≤ 0.05; **P ≤ 0.01.

We next detected the immunoregulatory function of *Id3*^*−/−*^ BMMSCs in NOD/ShiLtJ mice. We injected WT, *Id3*^*−/*−^ BMMSCs, or *Id3*^*−/−*^ BMMSCs that were pretreated with a BMP4 or COX2 inhibitor, into NOD/ShiLtJ mice at 16 weeks of age. No differences in body weights or submandibular gland weights between all NOD/ShiLtJ mouse groups (Supplementary Fig. 5A–C). WT BMMSCs-treated group exhibited improved salivary flow rate, reduced the area of inflammation and numbers of infiltrated CD4^+^ T cells and Th1 cells compared with untreated NOD/ShiLtJ mice 2 weeks after BMMSCs infusion (Fig. 5a–e). However, the salivary flow rate of *Id3*^*−/−*^ BMMSCs-treated mice was significantly higher than that of WT BMMSCs-treated mice (Fig. 5a). Consistent with the salivary flow rate improvement, *Id3*^*−/−*^ BMMSCs transplantation significantly reduced the area of inflammation in the submandibular glands compared with that in WT BMMSCs-treated NOD/ShiLtJ mice (Fig. 5b, c). Importantly, the improved therapeutic effects of *Id3*^−*/*−^ BMMSCs transplantation were completely abolished by pre-inhibition of BMP4 or COX2 with specific inhibitors (Fig. 5a–c). Moreover, the numbers of infiltrated CD4^+^ T cells and Th1 cells in the submandibular glands were decreased in the *Id3*^*−/−*^ BMMSCs-treated group compared with those in the WT BMMSCs-treated group, and these effects were blocked by pretreatment of *Id3*^−*/−*^ BMMSCs with a BMP4 or COX2 inhibitor (Fig. 5d, e). These data confirmed the biological significance of *Id3*^*−/−*^ BMMSCs-mediated immunosuppression of inflammation by upregulating BMP4 in an experimental SS model. Extending our findings in mice to humans, five pSS patients and five healthy volunteers were enrolled for bone marrow collection, after which their BMMSCs were cultured. BMP4 expression and PGE_2_ secretion were significantly decreased in BMMSCs from pSS patients compared with those from healthy volunteers (Fig. 6a–c).

## Discussion

The expansion of T cells plays an important role in many immune-related diseases, which can contribute to abnormal T-cell accumulation and chronic inflammation. Th1 cells produce high levels of IFN-γ, and are responsible for both phagocyte activation and production of opsonizing and complement-fixing antibodies^26^. They also contribute to the development of organ-specific autoimmune diseases, including SS, experimental autoimmune encephalomyelitis, collagen-induced arthritis, and inflammatory bowel disease^27^. In the present study, we showed that *Id3*^*−/−*^ BMMSCs suppressed T-cell proliferation and IFN-γ Th1 cells, which provided a novel cellular pathway to target in the treatment of immune-related diseases. Our finding that *Id3*^*−*/−^ BMMSCs exhibited a stronger immunoregulatory role than WT BMMSCs is less consistent with primary findings of SS-like inflammation in *Id3*^*−*/*−*^ mice^20^. This could be explained by the observation that T-cell dysfunction has a prominent role in the development of SS in *Id3*^*−/*−^ mice^20^. Several lines of evidence have supported this conclusion. For example, adoptive transfer of *Id3*^*−/−*^ T cells in mice is sufficient to induce symptoms of SS, but neonatal (3-day) thymectomy in *Id3*^*−*/*−*^ mice reduced these symptoms^20^ Moreover, conditional knockout mice with a specific deletion of *Id3* only in T cells show a similar SS phenotype as systemic *Id3*^*−*/*−*^ mice do^21^. The enhanced immunoregulatory role of BMMSCs in *Id3*^*−/−*^ mice may have been masked by the dominant immunopathogenic role of T cells in these mice. In this study, we also found *Id3* levels of BMMSCs in NOD/ShiLtJ mice were high expressed. As our results showed *Id3* depletion enhance the immunoregulatory role of BMMSCs, combining with our previous study have showed the impaired immunoregulatory role of BMMSCs in NOD/ShiLtJ mice, the increased *Id3* levels of BMMSCs would be a pathogenic factor induced a Sjögren’s syndrome. This is different to *Id3* knockout mouse, as there is different immunopathology between NOD/ShiLtJ mice and *Id3* knockout mice. In *Id3* knockout mouse model, there is no obvious change of Th1 cell related cytokine IFN-γ, IL-2, and TNFα, but have uncontrolled IL-4 production, which is the Th2 cell related cytokine^21^. This indicated that the Sjögren’s syndrome in Id3 knockout mouse was majorly involved by Th2 cells. However, the T cell involved in NOD/ShiLtJ mice was dominated by Th1 cell^1^. The different mechanism in the two models may lead to the different performance of immunoregulatory role of BMMSCs. Thus, our finding that ID3 exhibits a negative role in immunoregulation by MSCs expands our knowledge of this key molecule in regulating the complex network between immune cells and nonimmune cells, such as MSCs.

ID3 contrasting role in T cells and MSCs in the present study is similar to TWIST1 (another basic helix–loop helix protein). TWIST1 genes encode basic helix–loop-helix transcription factors involved in the expression of cytokine genes in inflammation^28^. TWIST1 regulates Th1-mediated acute and chronic inflammation. T cells isolated from chronically inflamed tissue with ulcerative colitis (UC) or Crohn’s disease (CD) and rheumatoid arthritis expressed high levels of TWIST1^29^. Mice deficient for TWIST1 have severe systemic inflammation, demonstrating the central role of TWIST1 proteins in the regulation of inflammation. However, depletion of TWIST1 significantly increased the secretion of PGE2, IDO1 and decreased IL-6 secretion in MSCs, and has enhanced suppressive function on proliferation of CD3^+^ T cells and expression of IFN-γ Th1. It also mediated IFN-γ regulated the anti-inflammatory and immuno-modulatory activities of MSCs^30^. The contrasted function of basic helix–loop–helix protein in MSCs and T cells demonstrating that its tissue specific function character.

PGE2 is the important soluble factors involved in exert their immunosuppressive effects of MSCs^31^. Our data showed that increased PGE2 was regulated by a novel ID3/BMP4/PGE_2_ axis in MSCs. As the expression of ID3 is reportedly regulated by BMP^32^, our findings first time provide important insights into the mechanisms through which ID3 regulates BMP4 via prevention of E2A binding to the *BMP4* promoter. Thus, our results revealed ID3 and BMP4 regulate each other’s expression. As a member of the transforming growth factor superfamily, BMP has important roles in immunity and in the development of autoimmune diseases^33^. For example, BMP4 signaling is involved in the differentiation and activation of natural killer cells^34^. BMP4 and BMP5 are downregulated in osteoarthritis, rheumatoid arthritis, and systemic lupus erythematous^35,36^. The expression of ID3 protein has been reported to be regulated by BMP. In this study, we showed that BMP4 plays an important role in immunoregulation of MSC and MSC-based therapy for experimental SS through induction of PGE_2_. This reveals a mutual regulation between ID3 and BMP4, this would be the molecular target by which host interact with MSCs immunoregulatory properties or MSC-based therapeutic effects. PGE_2_ is an important immune regulator^37,38^, and the decreased concentration of PGE_2_ is associated with the risk of developing an autoimmune disease, such as multiple sclerosis, Crohn’s disease, and multiple ulcerative colitis^39–42^. PGE_2_ can exert pro-inflammatory and anti-inflammatory effects depending on the context and target. The effects of PGE_2_ on CD4^+^ cells vary depending on the CD4^+^ T-cell subset, PGE_2_ concentration, and activation status of the cells^43^. Evidence has shown that PGE_2_ inhibits the proliferation of T cells and suppresses IFN-γ production from Th1 cells^44^. In addition, our previous work has demonstrated that periodontal ligament MSCs suppress T-cell proliferation via PGE_2_^45^. In our present study, we also showed PGE_2_ function in the SS therapy, these should have implications for treatment option for SS patients.

Although ID3 expression is not impaired in salivary glandular epithelial cells, peripheral T cells, or labial salivary glands in these patients^46^. Our data confirmed the biological significance decreased of BMP4 and PGE_2_ by MSCs in SS, demonstrating that BMP4/PGE_2_ can be the cellular pathway to target in the treatment of SS in human patients. These findings provide novel insights into the intrinsic mechanisms of MSC immunoregulation and may facilitate the development of novel therapies for SS and other immune diseases.

### Materials and methods

#### Study approval

Animal experiments with C57BL/6, BALB/cJ, NOD/ShiLtJ and/or *Id3*^*−/*−^ mice were approved by the Committee of the Capital Medical University (AEEI-2015-080), and experiments with E2a^f/f^Heb^f/f^ER-Cre^+^ and/or DO11.10 TCR-transgenic mice were approved by the Animal Care and Use Committees of the National Institute of Dental and Craniofacial Research (ASP18-860). The experiments with human samples were approved by the ethics committee of the Drum Tower Hospital of Nanjing University Medical School (2008017), and all human participants gave written informed consent.

### Mice

C57BL/6 mice (female, 6–8 weeks old), BALB/cJ mice (female, 6–8 weeks old), and NOD/ShiLtJ mice (female, 16 weeks old) were obtained from Jackson Laboratories. E2a^f/f^Heb^f/f^ER-Cre^+^ mice (on C57BL/6 mice background, female, 6–8 weeks old) and DO11.10 TCR-transgenic mice (on a BALB/cJ background, female, 6–8 weeks old) were purchased from Taconic (New York, USA). *Id3*^−*/*−^ mice (on C57BL/6 mice background) were bred under specific-pathogen-free conditions in the animal facility of the Capital Medical University.

### pSS patients

All patients had to meet the classification criteria based on the revised American-European criteria (2002) for primary Sjögren’s syndrome (pSS). In brief, these criteria comprise subjective criteria (ocular and oral symptoms) and objective criteria (ocular signs, histopathological signs [focus score ≥ 1] or positive parotid sialography, oral signs, and serological signs [presence of antinuclear antibodies, anti-SSA or anti-SSB antibodies]). pSS is diagnosed when 4 of the 6 aforementioned criteria are present, as long as histopathology or serology is positive, or when 3 of any 4 objective criteria are present. In addition, patients had to be informed of the investigational nature of this study. Exclusion criteria were (1) active, uncontrolled infections, and (2) end-stage organ failure.

### BMMSCs culture

Mouse bone marrow-derived mesenchymal stem cells (BMMSCs) were prepared from the bone marrow of femurs and tibias harvested from 6- to 8-week-old mice. Human BMMSCs were obtained from the iliac bone marrow. All BMMSCs cultured in Dulbecco’s modified Eagle’s medium with low glucose (DMEM-LG) containing 10% fetal bovine serum (FBS, Gibco). The BMMSCs were subsequently incubated at 37 °C in humid air with 5% CO_2_ and 20% O_2_. The medium was replaced every 3 days after the initial plating, and then replated at a dilution of 1:3 and third passage was used for experimentation. The stem cell properties of BMMSCs were characterized using cell surface markers (Sca-1, CD34, CD44, and CD45) and analyzed by flow cytometry. The pure rate of MSCs is almost 95% (Supplementary Fig. 2A).

### T-cell culture and activation

Splenocytes from 6- to 8-week-old mice were used to purify naive CD4^+^CD25^−^ T cells by using naive T cell isolation kit according to the manufacturer’s instructions (Miltenyi Biotec, Bergisch Gladbach, Germany). For all T cells cocultured with BMMSCs from WT mice, *Id3*^*−/*−^ mice and human, the medium contained beads coated with anti-CD3 and anti-CD28 antibodies for T-cell activation. CD4^+^CD25^−^ T cells were cocultured with BMMSCs at a ratio of 1:1, 2:1, or 4:1. For blocking assay, BMMSCs from *Id3*^−*/*−^ mice before coculture were pretreated COX2 inhibitor (celecoxib, 5 μmol/5 × 10^5^ cells; Selleck), TNF-α inhibitor (pomalidomide, 0.5 μmol/5 × 10^5^ cells; Selleck) or neutralizing anti-IL-10 antibody (20 μg/ml/5 × 10^5^ cells; PeproTech).

### T-cell proliferation assay

Mouse naive (CD4^+^CD25^*−*^) T cells were resuspended in rewarmed phosphate-buffered saline (PBS) at a final concentration of 5 × 10^6^ cells/mL then labeled with 5 mM carboxyfluorescein succinimidyl ester (Invitrogen) according to the manufacturer’s instructions. T cells were cocultured with BMMSCs from wild-type (WT) or *Id3*^−*/−*^ mice at a ratio of 1:2 for 4 days. Lymphocytes were then harvest and assayed by flow cytometry. The proliferation index was calculated using ModFit LT 3.0 software (Verity Software House, USA) as the average number of cell divisions.

### In vivo induction of ovalbumin-specific T cells

1 × 10^6^ CD4^+^KJ-126^+^CD25^*−*^ naive T cells isolated from DO11.10 TCR-transgenic mice were injected i.v. through caudal vein into BALB/cJ mice, together with 5 × 10^5^ BMMSCs from WT or *Id3*^−/−^ mice. After 4 h, ovalbumin (50 μg) in complete Freund’s adjuvant solution (25 μL) combined with 25 μL saline was injected into the footpad of BALB/cJ mice. Mice were euthanized 7 days later, and T cells in the draining lymph nodes were analyzed by fluorescence-assisted cell sorting.

### BMMSCs transfection

BMMSCs (1 × 10^5^ per well) were cultured overnight in 24-well plates and transfected with 40 nM or 75 nM of microRNA (miRNA) negative control (NC), or 50 nM or 100 nM miRNA inhibitor to knock down miRNA expression. To knock down gene expression, BMMSCs were transfected with 100 nM negative siRNA (Ctrl) or gene specific siRNA using Lipofectamine RNAiMAX (Invitrogen). The shRNA were purchased (Lce1h shRNA, TG503987, OriGene, Maryland, USA; Serpinb2 shRNA, sc-40805-SH, Santa Cruz Biotechnology). The siRNA of Bmp4, Cxcl12, E2A were purchased (Bmp4 and Cxcl12 from RiboBio Guangzhou, China; E2A from Santa Cruz Biotechnology). The nucleotide sequences of other siRNA are listed in Supplementary Methods.

### Overexpressing plasmid construction and viral infection

Mouse full-length Id3 and E2A cDNA were constructed with standard methods and fused to a Hemagglutinin (HA) tag and M2-FLAG tag respectively, was produced. This sequence (HA-Id3 and Flag-E2A) was subcloned into the pQCXIN retroviral vector with AgeI and BamH1 restriction sites. For viral infections, BMMSCs form WT mice were plated overnight, then infected with retroviruses in the presence of polybrene (6 µg/mL, Sigma-Aldrich, St. Louis, MO, USA) for 6 h. After 48 h, infected BMMSCs were selected with different antibiotics. Ectopic Id3 and E2A overexpression in transduced BMMSCs were confirmed by Western Blot analysis.

### Co-immunoprecipitation (Co-IP) assay

The assay was detected by HA-Tag Co-IP kit (Thermo Fisher Scientific, Waltham, MA, USA) according to the manufacturer’s protocol. WT mice BMMSCs transfected with vector plasmid or HA-Id3 plasmid were washed carefully with pre-chilled PBS two times; then, cold RIPA lysis buffer was added and cell lysate was collected. Ten microliters of anti-HA agarose slurry was added into each labeled spin column with gentle end-over-end mixing at 4 °C for overnight. Then wash with TBS-T and Elution Buffer (10 μL) was added to the anti-c-HA agarose. At last the sample was heated for 10 min at 95–100 °C and the supernatant collected for western blot analysis.

### Allogeneic BMMSC transplantation following BMP4 or COX2 inhibition

Before blocking, BMMSCs from *Id3*^−*/*−^ mice were washed twice with PBS. BMP4 inhibitor (LDN-193189, 40 nmol/5 × 10^5^ cells; Selleck, Texas, USA), COX2 inhibitor (celecoxib, 5 μmol/5 × 10^5^ cells; Selleck) were added and incubated at 37 °C for 12 h. Then, cells were harvested, washed twice with PBS and prepared for infusion. According to our previous study, 16-week-old female NOD/ShiLtJ mice were randomization divided into control groups, WT BMMSCs injection, WT BMMSCs injection with BMP4 inhibition, Id3 KO BMMSCs injection, Id3 KO BMMSCs injection with BMP4 or COX2 inhibition (*n* = 10 in each group). For BMMSC transplantation, each group NOD/ShiLtJ mice were injected with BMMSCs (1 × 10^6^ cells/mouse) in 0.15 mL PBS or only 0.15 mL PBS via their tail vein.

### Salivary flow rate

To assess the salivary flow rate, NOD/ShiLtJ mice were weighed, and mild anesthesia was induced with a solution of ketamine (100 mg/mL; Beijing Double-Crane Pharmaceutical Co., China) and xylazine (20 mg/mL; Sigma, St. Louis, MO, USA) in sterile water, given intraperitoneally (1 μL/g body weight). Salivary secretion was stimulated using 0.1 mL/kg body weight of a pilocarpine solution (50 mg/mL; Beijing Double-Crane Pharmaceutical Co.) subcutaneously. Saliva collection began within 2 min of pilocarpine administration. Animals were positioned with a 75-mm hematocrit tube placed in the oral cavity, and whole saliva was collected into pre-weighed 0.75-mL tubes for 10 min. The amount of saliva collected was determined gravimetrically.

### Preparation of cells from submandibular glands

NOD/ShiLtJ mice were anesthetized with a 260-µL ketamine/xylazine/acepromazine mixture (2 mg ketamine, 0.4 mg xylazine and 60 µg acepromazine in PBS) by intraperitoneal injection, and the submandibular glands were harvested and washed with ~5 mL PBS to remove circulating blood. The glands were cut into small pieces and digested twice at 37 °C for 20 min each with complete RPMI containing 2.4 mg/mL collagenase type I (Gibco/Invitrogen, Basel, Switzerland) and 0.2 mg/mL DNase I (Roche Diagnostics, Rotkreuz, Switzerland). Mononuclear cells were purified by 30%-Percoll gradient centrifugation at 1600 r/min (Solarbio, Beijing, China). Complete RPMI consisted of RPMI 1640 medium (Invitrogen) with 10% fetal calf serum (Omnilab, Mettmenstetten, Switzerland), 2 mM l-glutamine (Gibco), and 1% penicillin-streptomycin (Gibco).

### T-cell staining and flow cytometry

Intranuclear staining was carried out with Fixation/Permeabilization buffer solution (eBioscience) according to the manufacturer’s instructions. For intracellular cytokine staining, cells were stimulated with phorbol myristate acetate (10 ng/mL), ionomycin (250 ng/mL) and Golgi-Plug (1:1000 dilution; BD Biosciences, New Jersey, USA) at 37 °C for 4 h, and then fixed with Fixation/Permeabilization buffer solution (BD Biosciences) according to the manufacturer’s instructions. Stained cells were analyzed on a FACSCalibur or LSRFortessa (both from BD Biosciences), and data were analyzed with FlowJo software (BD Biosciences).

### Antibodies

Purified anti-mouse CD3 antibody (no azide/low endotoxin; 145-2C11), purified anti-mouse CD28 antibody (no azide/low endotoxin; 37.51), and fluorochrome-conjugated antibodies (anti-mouse CD4 [RM4-5], anti-mouse CD8α [53–6.7], anti-mouse CD45 [30-F11], anti-mouse CD25 [PC61.5 and eBio7D4], anti-mouse DO11.10 TCR [KJ1-26], anti-mouse/rat Foxp3 [FJK-16a], anti-mouse IL-4 [11B11], and anti-mouse IL-13 [eBio13A]) were purchased from eBioscience (California, USA). Fluorochrome-conjugated anti-mouse IL-17A (TC11-18H10.1), anti-mouse IFN-γ (XMG1.2) and anti-mouse IL-10 antibody (JES5-16E3) were purchased from BioLegend (California, USA).

### Total RNA extraction and microarray assay

BMMSCs from WT mice and *Id3*^*−/*−^ mice were seeded in 10-cm^2^ dishes, cultured until they reached 80% confluence. Total RNA was extracted using TRIzol Reagent (Life Technologies, California, USA). Following purification with an RNeasy kit (Qiagen, Valencia, CA, USA), cDNA was generated using One-Cycle Target Labeling and Control Reagents (Affymetrix, Santa Clara, CA, USA), and cRNA was created with a GeneChip IVT Labeling Kit (Affymetrix). For more details, see Supplementary Methods.

### Quantitative real-time reverse transcription polymerase chain reaction (qRT-PCR)

Total RNA was extracted from cells using TRIzol Reagent (Invitrogen) and reverse transcribed to cDNA using a PrimeScript RT Reagent Kit (Takara, Dalian, China). Amplification of target genes was performed by qPCR using the cDNA as a template, specific primers, and a SYBR PrimeScript RT-PCR Kit (Takara) on an ABI PRISM 7900 Real-Time PCR System (Applied Biosystems, Carlsbad, CA, USA). PCR amplification was performed in duplicate at 95 °C for 15 s, followed by 40 cycles at 95 °C for 5 s, at 60 °C for 30 s, at 95 °C for 15 s, at 60 °C for 15 s, and at 95 °C for 15 s. The specific primers for qPCR of the gene region were showed in Supplementary Methods.

### Western blotting

BMMSCs (2 × 10^6^/tube) were lysed in RIPA Lysis and Extraction Buffer (Thermo Fisher Scientific, Rockford, IL, USA). Individual cell lysates (10 μg/lane) were separated by sodium dodecyl sulfate polyacrylamide gel electrophoresis and transferred to Immobilon-P polyvinylidene difluoride membranes (Millipore, Billerica, MA, USA). After blocking with SuperBlock T20 (PBS) Blocking Buffer (Thermo Fisher Scientific), the membranes were incubated with rabbit monoclonal antibody against BMP4 (ab39973, Abcam, Cambridge, UK, 1:1000 dilution), rabbit monoclonal antibody against COX2 (12282, Cell Signaling Technology, Massachusetts, USA, 1:1000 dilution), rabbit polyclonal antibody against ID3 (BCH-4/17-3, Biocheck, California, USA, 1:1000 dilution), rabbit monoclonal antibody against E2A (sc-365261, Santa Cruze, California, USA, 1:1000 dilution). Bound antibodies were detected with horseradish peroxidase-conjugated secondary antibodies (Santa Cruze, California, USA, diluted 1:10,000) and visualized using Pierce ECL Western Blotting Substrate (Thermo Fisher Scientific), followed by exposure of the membranes to film and digital imaging (Bio-Rad, California, USA).

### Chromatin immunoprecipitation assay

To analyze promoter binding, chromatin immunoprecipitation assays were carried out. Briefly, cells were collected, fixed at room temperature with 1% formaldehyde for 10 min, and lysed in lysis buffer (Diagenode, New Jersey, USA). Lysates were sonicated and then precipitated with anti-E2A antibody (V-18; Santa Cruz Biotechnology, Santa Cruz, CA, USA), anti-ID3 antibody (BCH-4/17-3; BioCheck, Foster City, CA, USA), control rabbit IgG (ab171870; Abcam, Cambridge, UK), or control mouse IgG (107.3; BD Biosciences). Chromatin immunoprecipitated DNA was analyzed by qPCR (Bio-Rad, Hercules, CA, USA) with the following primers: forward 5′-AGGCCACCCTTTAAACCAAT-3′; reverse 5′-AATCACCATTTACCCCGAGT-3′.

### Histological analysis of submandibular glands

Submandibular gland samples were fixed with 4% paraformaldehyde at 4 °C for 24 h, embedded in paraffin, and sliced into 5-μm thick sections. After hematoxylin/eosin (H&E) staining, sections were photographed at room temperature using a microscope (Olympus BX51, Japan), and the area of inflammatory foci (containing ≥50 lymphocytes per 4 mm^2^ tissue) was calculated per field at ×200 magnification (×20 objective lens) using Image-Pro Plus software v6.0 (Media Cybernetics, Maryland, USA). Five entire salivary gland sections for each animal were counted, with an average of 10 fields/gland, by an experienced expert in histopathology in a blinded fashion.

### Statistics

All experiments are randomized into groups of similar sample size by block randomization. Data collection and analysis were performed blindly. No samples and animals were excluded from analysis. All experiments were performed at least in triplicates. Comparisons between two groups were performed using unpaired two-tailed Student’s *t* tests; one-way analysis of variance (ANOVA; with Tukey’s multiple comparison post hoc test, Post tests were run only if F achieved *P* < 0.05.) was used for comparisons between more than two groups. *P* values < 0.05 were considered statistically significant. Statistical analyses were carried out using GraphPad Prism 6 (California, USA). Statistical power analysis was used to ensure adequate sample size for detecting significant difference between samples. The variance is similar between groups that are being statistically compared.

## Supplementary information

Supplemental figure 1

Supplemental figure 2

Supplemental figure 3

Supplemental figure 4

Supplemental figure 5

Supplemental figure legend

Supplemental Table
