# Supplementary figures and images for: Depletion of ID3 enhances mesenchymal stem cells therapy by targeting BMP4 in Sjögren’s syndrome
Source: Cell Death Dis. 2020 Mar 5;11(3):172. doi: 10.1038/s41419-020-2359-6 (PMC7058624; doi:10.1038/s41419-020-2359-6)

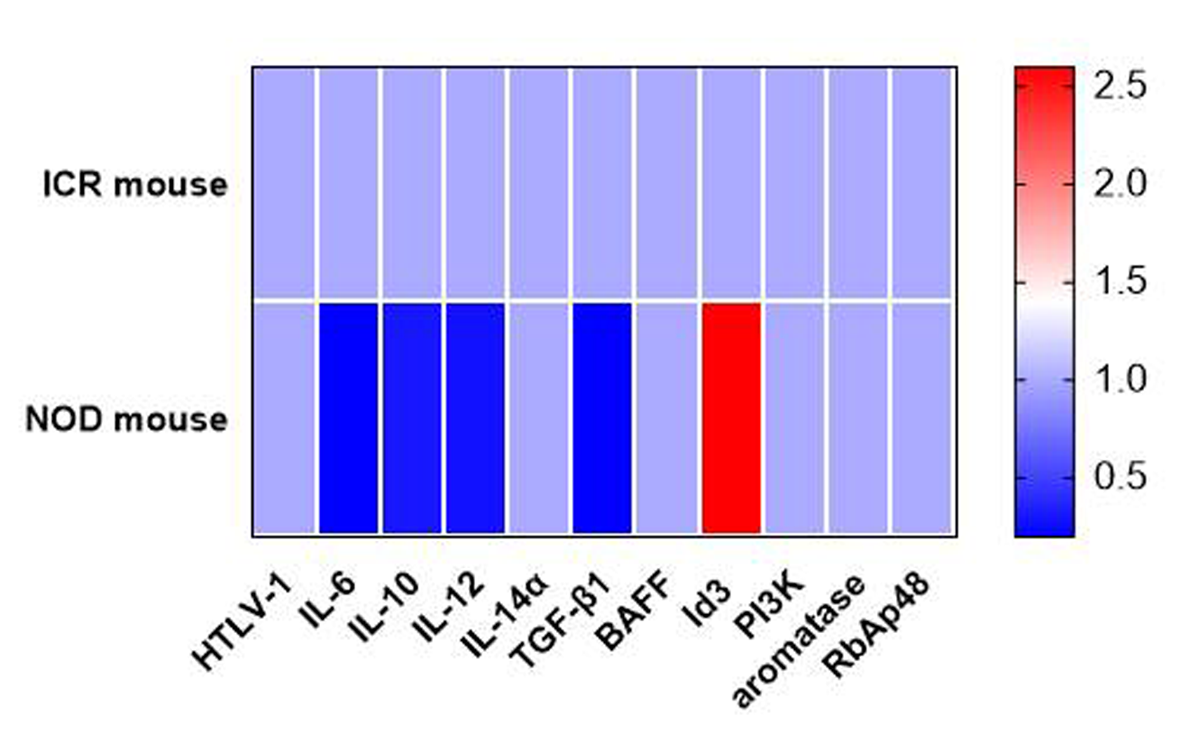

Supplement: Supplementary file 1 — Supplemental figure 1 [file 41419_2020_2359_MOESM1_ESM.tif]

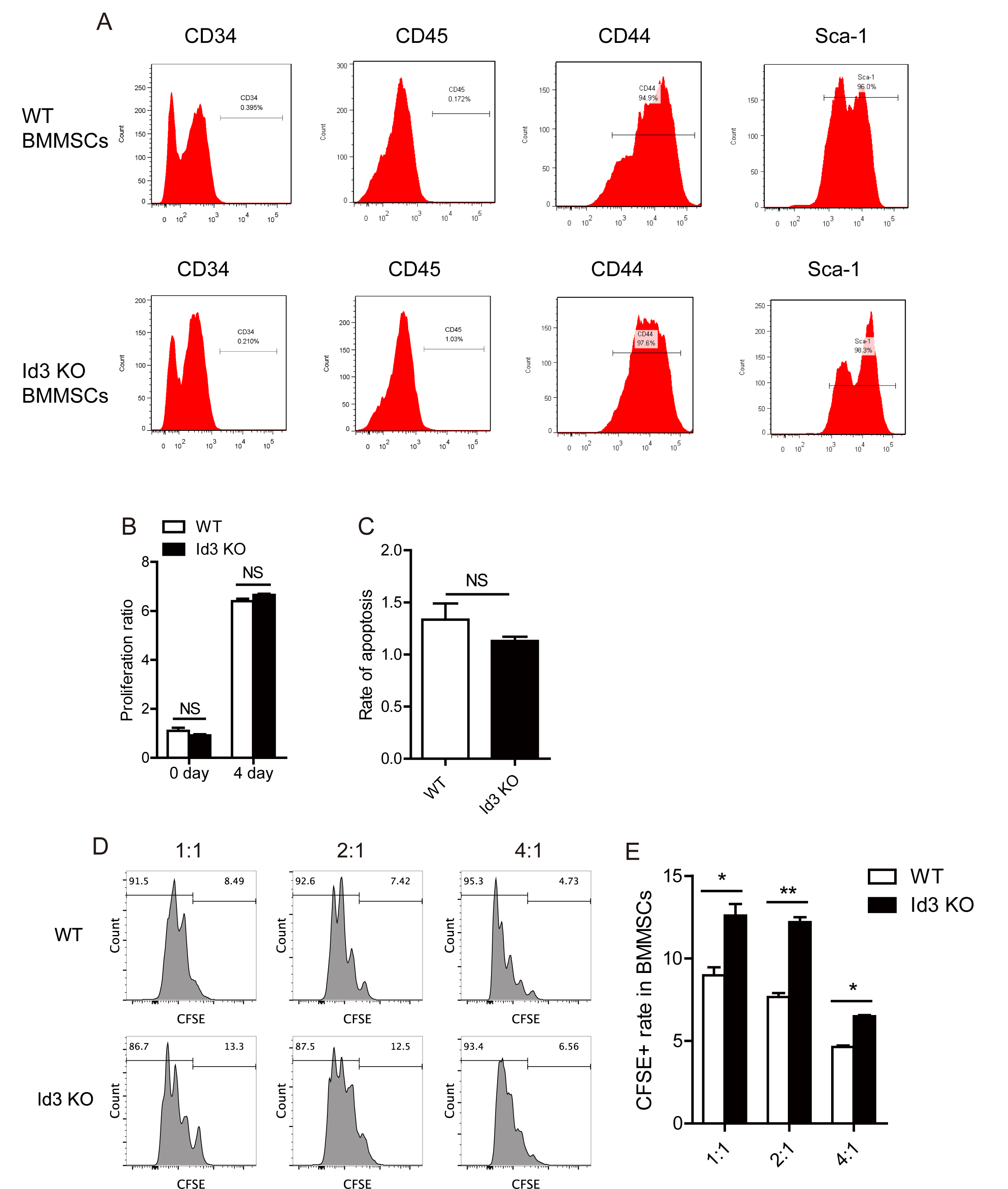

Supplement: Supplementary file 2 — Supplemental figure 2 [file 41419_2020_2359_MOESM2_ESM.tif]

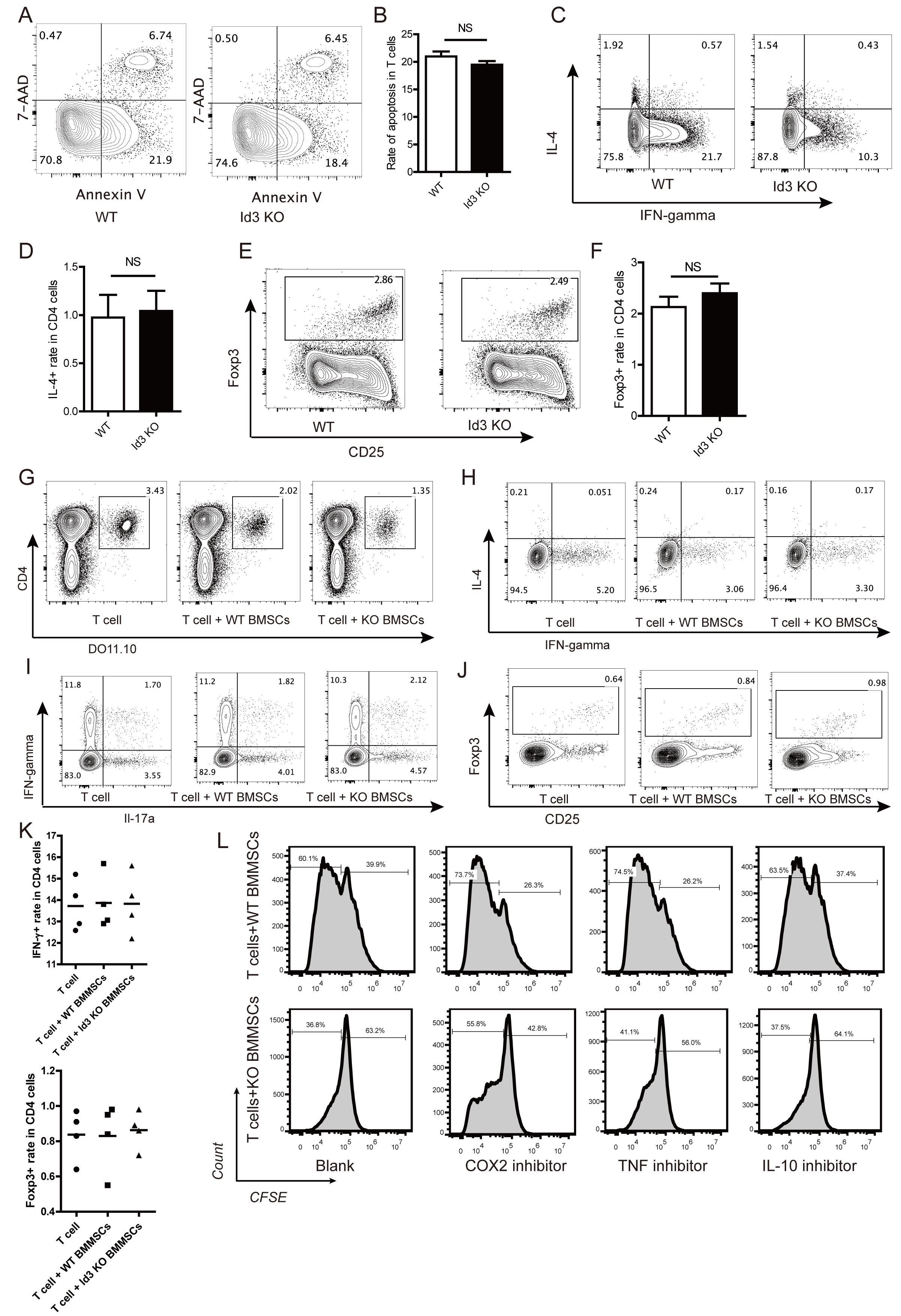

Supplement: Supplementary file 3 — Supplemental figure 3 [file 41419_2020_2359_MOESM3_ESM.tif]

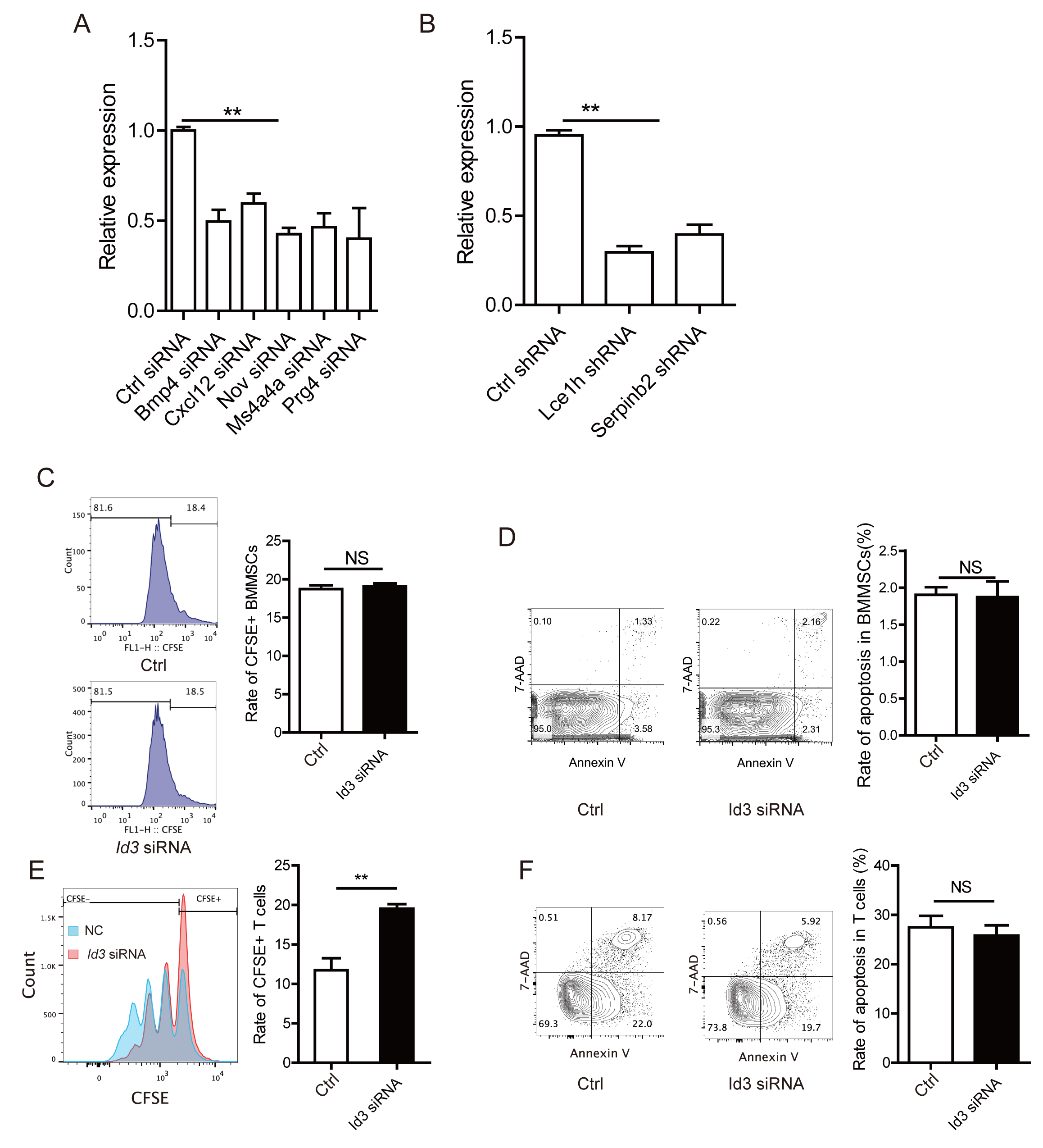

Supplement: Supplementary file 4 — Supplemental figure 4 [file 41419_2020_2359_MOESM4_ESM.tif]

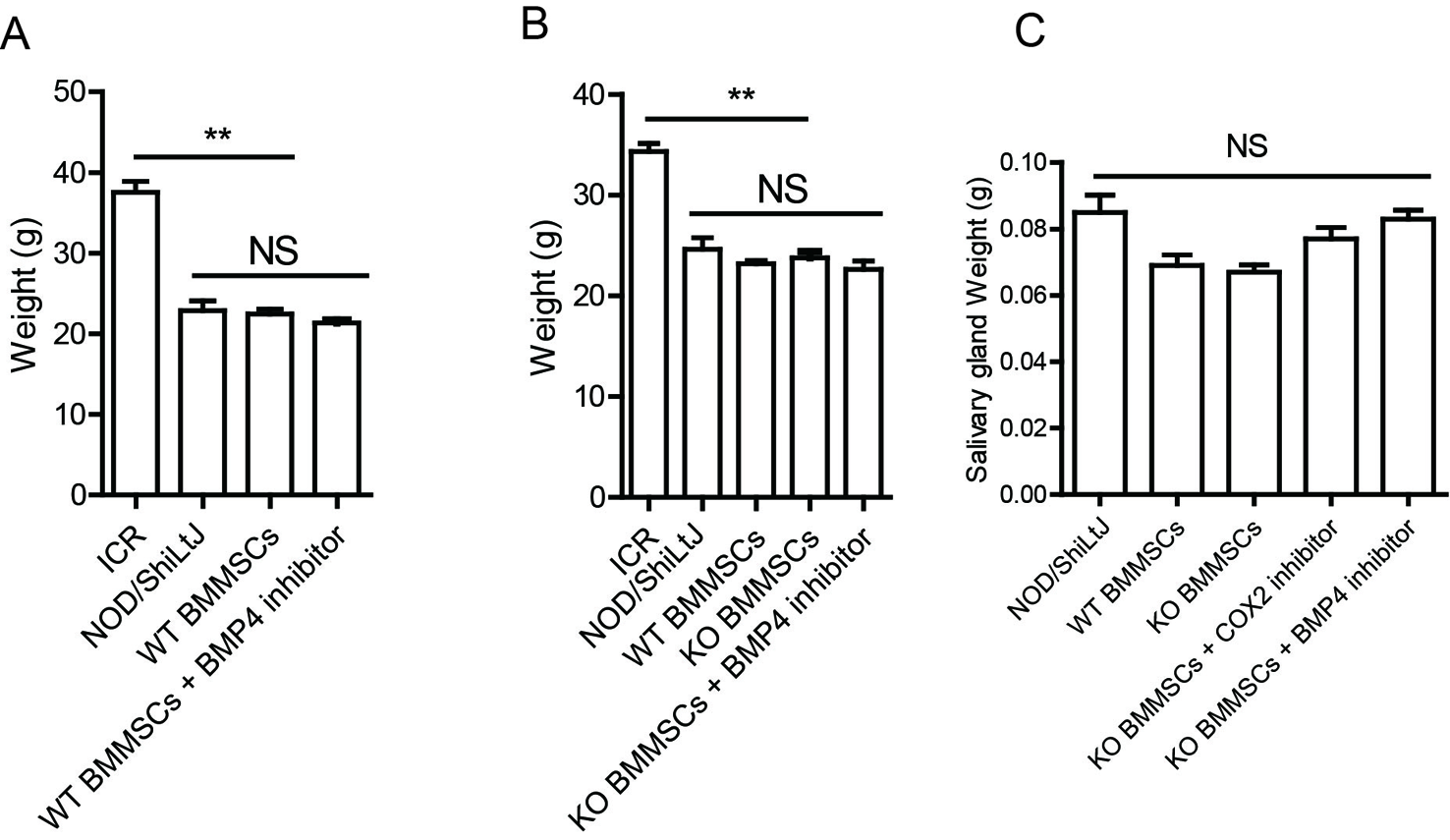

Supplement: Supplementary file 5 — Supplemental figure 5 [file 41419_2020_2359_MOESM5_ESM.tif]
