# Supplementary material for: Depletion of ID3 enhances mesenchymal stem cells therapy by targeting BMP4 in Sjögren’s syndrome
Source: Cell Death Dis. 2020 Mar 5;11(3):172. doi: 10.1038/s41419-020-2359-6 (PMC7058624; doi:10.1038/s41419-020-2359-6)
Supplement: Supplementary file 6 — Supplemental figure legend [file 41419_2020_2359_MOESM6_ESM.docx]

**Supplemental Figure legends**

**Supplemental Figure 1. *Id3* was upregulated in BMMSC of NOD/ShiLtJ mice compared with ICR mice**

Gene array were used to compare the gene expression in BMMSC between NOD/ShiLtJ mice and ICR mice. Among the reported SS related genes in mice models, Id3 is the highest expressed gene.

**Supplemental Figure 2. *Id3* depletion did not influence BMMSC proliferation or apoptosis**

(A): The flow cytometry showed WT BMMSCs and Id3 knockout BMMSCs positive expressed CD44 and Sca-1, negative expressed CD34 and CD45. The pure rate of MSCs is almost 95%. (B): Carboxyfluorescein succinimidyl ester (CFSE) analyses of the effects of *Id3* knockout on the proliferation of BMMSCs after 4 days of culture. (C): Annexin V/PI analyses apoptosis of WT BMMSCs and *Id3^-/-^* BMMSCs. (D): Carboxyfluorescein succinimidyl ester analyses the immunosuppression on T cells after cocultured with WT BMMSCs and *Id3^-/-^* BMMSCs at 1:1, 2:1, 4:1. (E): Quantitative analyses of CFSE positive T cells. (n=5, three independent experiments). Values are means ± SD. Student’s t tests and one-way ANOVA. **P* ≤ 0.05; ***P* ≤ 0.01.

**Supplemental Figure 3. Flow cytometry analyses of T cells cocultured with WT BMMSCs or *Id3^-/-^* BMMSCs**

(A, B): FCS data for T-cell apoptosis after culture with *Id3^-/-^* mice BMMSCs or WT mice BMMSCs. FCS data of the frequencies of Th2 (C, D) and Treg subsets (E, F). (G–J): FCS data showing changes in Th1, Th2 and Treg subsets following injection in vivo. (K): Changes in Tregs and Th1 cells in each group. (L): FCS data of T-cell proliferation after culture with BMMSCs treated with COX2, TNF-α, or IL-10 inhibitors. (n=5, three independent experiments). Values are means ± SD. Student’s t tests and one-way ANOVA. **P* ≤ 0.05; ***P* ≤ 0.01.

**Supplemental Figure 4. *Id3* knockdown by siRNA in BMMSCs resulted in phenotypes similar to those in *Id3^-/-^* BMMSCs**

(A): qPCR analyses the *Bmp4, Cxcl12, Nov, Ms4a4a and Prg4* knockdown efficiency by siRNA, negative control siRNA transfected as control group. (B): qPCR analyses the *Lce1h and Serpinb2* knockdown efficiency by shRNA, negative control shRNA transfected as control group. (C): Carboxyfluorescein succinimidyl ester analyses of the effects of Id3 siRNA on the proliferation of BMMSCs, (D): FCS analyses of apoptosis of BMMSCs. (E, F): Effects of BMMSCs transfected with Id3 siRNA on T-cell proliferation and apoptosis. (n=5, three independent experiments). Values are means ± SD. Student’s t tests and one-way ANOVA. **P* ≤ 0.05; ***P* ≤ 0.01.

**Supplemental Figure 5. Changes in mouse body and salivary gland weights among groups**

(A): Body weights of mice in the NOD/ShiLtJ, WT BMMSCs, and WT BMMSCs with BMP4 inhibitor groups. (B): Body weights of mice in the NOD/ShiLtJ, WT BMMSCs, Id3 KO BMMSCs, and Id3 KO BMMSCs with BMP4 inhibitor groups. (C): Salivary gland weights of mice in the NOD/ShiLtJ, WT BMMSCs, Id3 KO BMMSCs, Id3 KO BMMSCs with COX2 inhibitor groups, and Id3 KO BMMSCs with BMP4 inhibitor groups. (n=10, three independent experiments). Values are means ± SD. Student’s t tests and one-way ANOVA. **P* ≤ 0.05; ***P* ≤ 0.01.
