# Supplementary material for: Depletion of ID3 enhances mesenchymal stem cells therapy by targeting BMP4 in Sjögren’s syndrome
Source: Cell Death Dis. 2020 Mar 5;11(3):172. doi: 10.1038/s41419-020-2359-6 (PMC7058624; doi:10.1038/s41419-020-2359-6)
Supplement: Supplementary file 7 — Supplemental Table [file 41419_2020_2359_MOESM7_ESM.docx]

**Supplemental Table**

Table Top 10 up-regulated and down-regulated genes (Id3 KO BMMSCs VS WT BMMSCs)

| Top 10 up-regulated genes | | | |  | Top 10 down-regulated genes | | | |
| --- | --- | --- | --- | --- | --- | --- | --- | --- |
| probe_id | symbol | pvalue | Log2FC |  | probe_id | symbol | p-value | Log2FC |
| 10357870 | Prelp | 2.23E-09 | 2.887156 |  | 10499932 | Lce1h | 4.90E-08 | -3.44779 |
| 10374777 | Efemp1 | 9.41E-07 | 2.112947 |  | 10557470 | Gdpd3 | 1.35E-06 | -2.47519 |
| 10474229 | Cd59a | 0.001339 | 2.024747 |  | 10473322 | Cwc22 | 2.75E-07 | -2.25305 |
| 10541075 | Cxcl12 | 1.01E-07 | 2.010626 |  | 10349157 | Serpinb2 | 5.57E-05 | -2.19584 |
| 10358476 | Prg4 | 5.11E-08 | 1.911498 |  | 10518526 | Angptl7 | 5.21E-08 | -2.15502 |
| 10424119 | Nov | 3.66E-06 | 1.814526 |  | 10499937 | Lce1j | 7.72E-05 | -1.52555 |
| 10461587 | Ms4a4a | 0.000785 | 1.513099 |  | 10455015 | Vaultrc5 | 0.000196 | -1.27892 |
| 10419261 | Bmp4 | 1.80E-05 | 1.440048 |  | 10379511 | Ccl2 | 8.38E-07 | -1.2007 |
| 10570957 | Sfrp1 | 5.40E-05 | 1.373799 |  | 10499935 | Lce1i | 0.000952 | -1.19074 |
| 10436304 | Abi3bp | 0.000108 | 1.284044 |  | 10549041 | Slco1a5 | 4.17E-05 | -1.18953 |
